# Synthesis and structure of 2,4-bis­(2,6-dimethyl-4*H*-pyran-4-yl­idene)-3-oxo­penta­nedi­nitrile, an unexpected product of a Knoevenagel condensation reaction

**DOI:** 10.1107/S2056989026000587

**Published:** 2026-01-23

**Authors:** Antonio Carella, Emmanuele Parisi, Vincenzo Piccialli, Roberto Centore

**Affiliations:** a Dipartimento di Scienze Chimiche, Università degli Studi di Napoli ’Federico II’, Complesso di Monte S. Angelo, Via Cinthia, 80126 Napoli, Italy; b Department of Applied Sciences and Technology, Polytechnic of Turin, Corso Duca degli Abruzzi 24, I-10129 Turin, Italy; University of Aberdeen, United Kingdom

**Keywords:** crystal structure, mol­ecular structure, Knoevenagel condensation

## Abstract

The title com­pound, C_19_H_16_N_2_O_3_, was the unexpected product of the Knoevenagel condensation between 2,6-dimethyl-γ-pyrone and cyano­acetic acid in the presence of acetic anhydride and piperidine as catalyst. The mol­ecule is formed by two almost planar 2,6-dimethyl-4*H*-pyran-4-cyanoyl­idene halves connected by the central carbonyl group. The two halves are not coplanar, the dihedral angle between their planes being 47.51 (3)°. The packing shows the formation of (001) sheets of mol­ecules held together by weak C—H⋯N and C—H⋯O hy­dro­gen bonds. A mechanism for the reaction is proposed.

## Abstract 2

The crysstal structure of an unexpected product of the Knoevenagel condensation between 2,6-dimethyl-γ-pyrone and cyano­acetic acid in the presence of acetic anhydride and piperidine as catalyst is reported and a mechanism for the reaction is proposed.

## 1. Chemical context

The Knoevenagel condensation is a classic organic reaction where an aldehyde or ketone reacts with a com­pound con­taining an activated methyl­ene group, *i.e.* a CH_2_ group linked to electron-withdrawing groups in the presence of a weak base as the catalyst or in a dehydrating environment (for reviews, see Jones, 1967 ▸; Heravi *et al.*, 2020 ▸). A new carbon–carbon bond is formed, resulting in an α,β-unsaturated com­pound after dehydration. This reaction is widely used in the synthesis of *n*-type organic semiconductors to introduce terminal electron-acceptor groups into the mol­ecular backbone (Fusco *et al.*, 2021 ▸; Fusco *et al.*, 2022 ▸, Yao *et al.*, 2023 ▸) and in the syn­thesis of organic sensitizers for Dye Sensitized Solar Cells (DSSC) (Yahya *et al.*, 2021 ▸; D’Amico *et al.*, 2023 ▸). In the latter case, typically, an aldehydic precursor is condensed with cyano­acetic acid to introduce a cyano­acrylic functionality essential to anchor the dye on a TiO_2_ mesoporous layer through the carb­oxy­lic acid group. Some of us reported on pyran-based organic sensitizers for DSSC, whose structure was based on a pyran electron-acceptor core symmetrically linked to two carbazole donor moieties and end capped with cyanoacrylic acid groups (Bonomo *et al.*, 2020 ▸). The studied dyes differ with respect to the electron-acceptor groups fun­c­tionalizing the pyran core: by reacting commercial 2,6-di­methyl-γ-pyrone with four different mol­ecules containing activated methyl­ene groups in a Knoevenagel condensation, four different electron-acceptor groups were linked to the pyran core. Following the same synthetic strategy, we tried to react the commercial pyran­one derivative with cyano­acetic acid to introduce a cyano­acrylic acid functionality directly on the pyran core. To our surprise, the main obtained product was not that expected, but 2,4-bis­(2,6-dimethyl-4*H*-pyran-4-yl­idene)-3-oxo­penta­nedi­nitrile, **CC** (Scheme 1), resulting from a more com­plex reaction involving two mol­ecules of 2,6-dimethyl-γ-pyrone and two of cyano­acetic acid. In this article, we report a full mechanistic and structural analysis of this new com­pound.
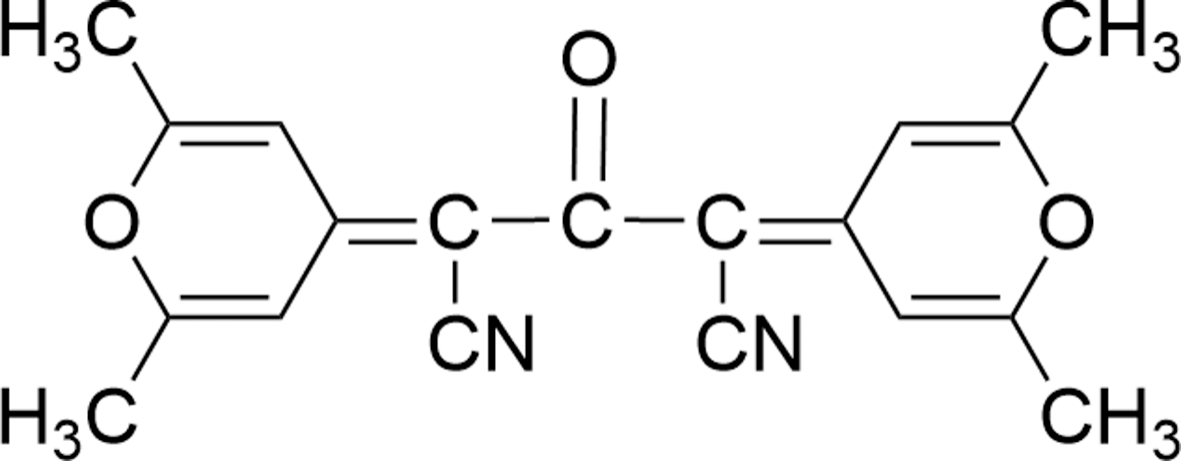


## 2. Structural commentary

**Figure 1:**
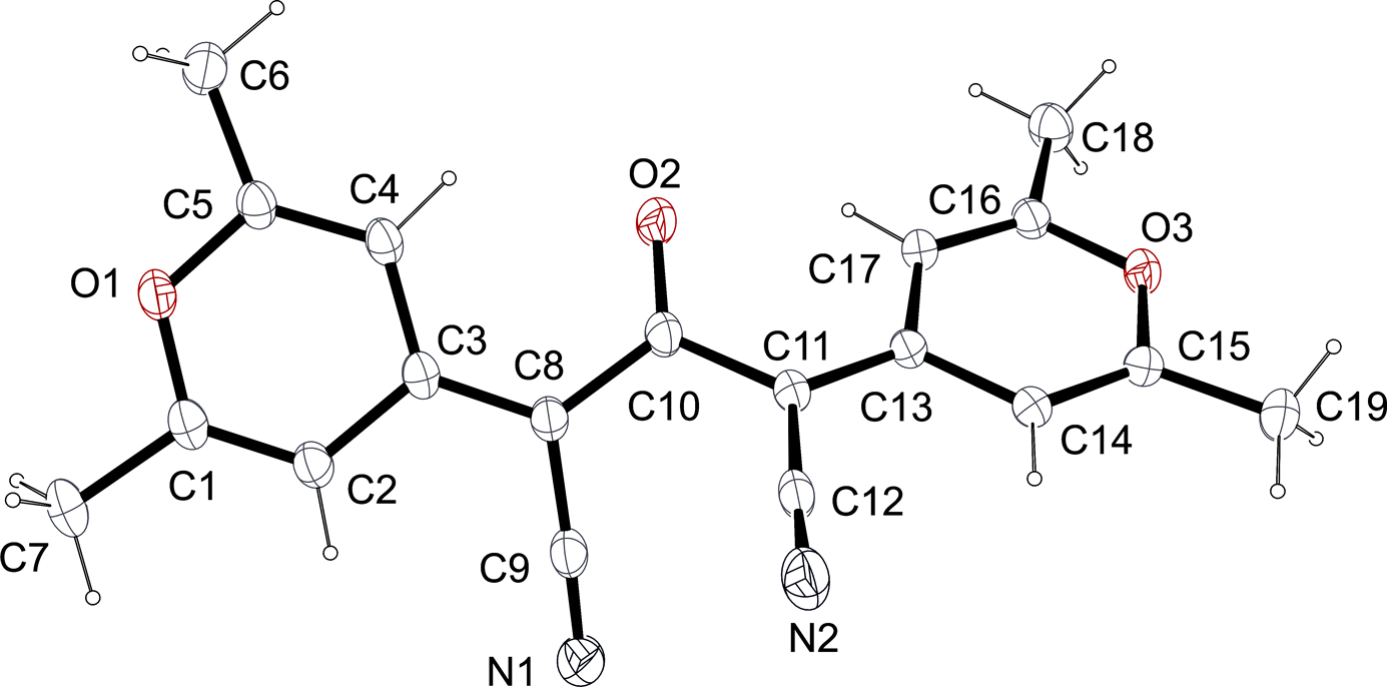
The mol­ecular structure of **CC**, with displacement ellipsoids drawn at the 30% probability level.

The mol­ecular structure of **CC**, which crystallizes in the triclinic space group *P*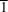 with one mol­ecule in the asymmetric unit, is shown in Fig. 1 ▸. The mol­ecule is formed from two 2,6-dimethyl-4*H*-pyran-4-cyanoyl­idene (C_9_H_8_N_2_O_2_) halves con­nected to the central C10=O2 carbonyl group. The two halves (H atoms excluded) are close to being planar [within 0.070 (2) Å] and the pattern of bond lengths clearly evidences π-conjugation, for example, C11—C10 = 1.486 (2) Å, C11—C13 = 1.385 (2) Å and C11—C12 = 1.426 (2) Å. However, the two halves are not coplanar, their least-squares planes making a dihedral angle of 47.51 (3)°. This dihedral angle is basically the result of twists around the C8—C10 and C10—C11 bonds [C3—C8—C10—O2 = −23.9 (3)° and O2—C10—C11—C13 = −26.7 (3)°]. These twists seem mainly due to the need to relax the close contact between the C atoms of the two cyano groups [C9⋯C12 = 2.830 (2) Å]. Two short intra­molecular C—H⋯O contacts (Table 1 ▸) to the central ketone O atom occur.

## 3. Supra­molecular features

**Figure 2:**
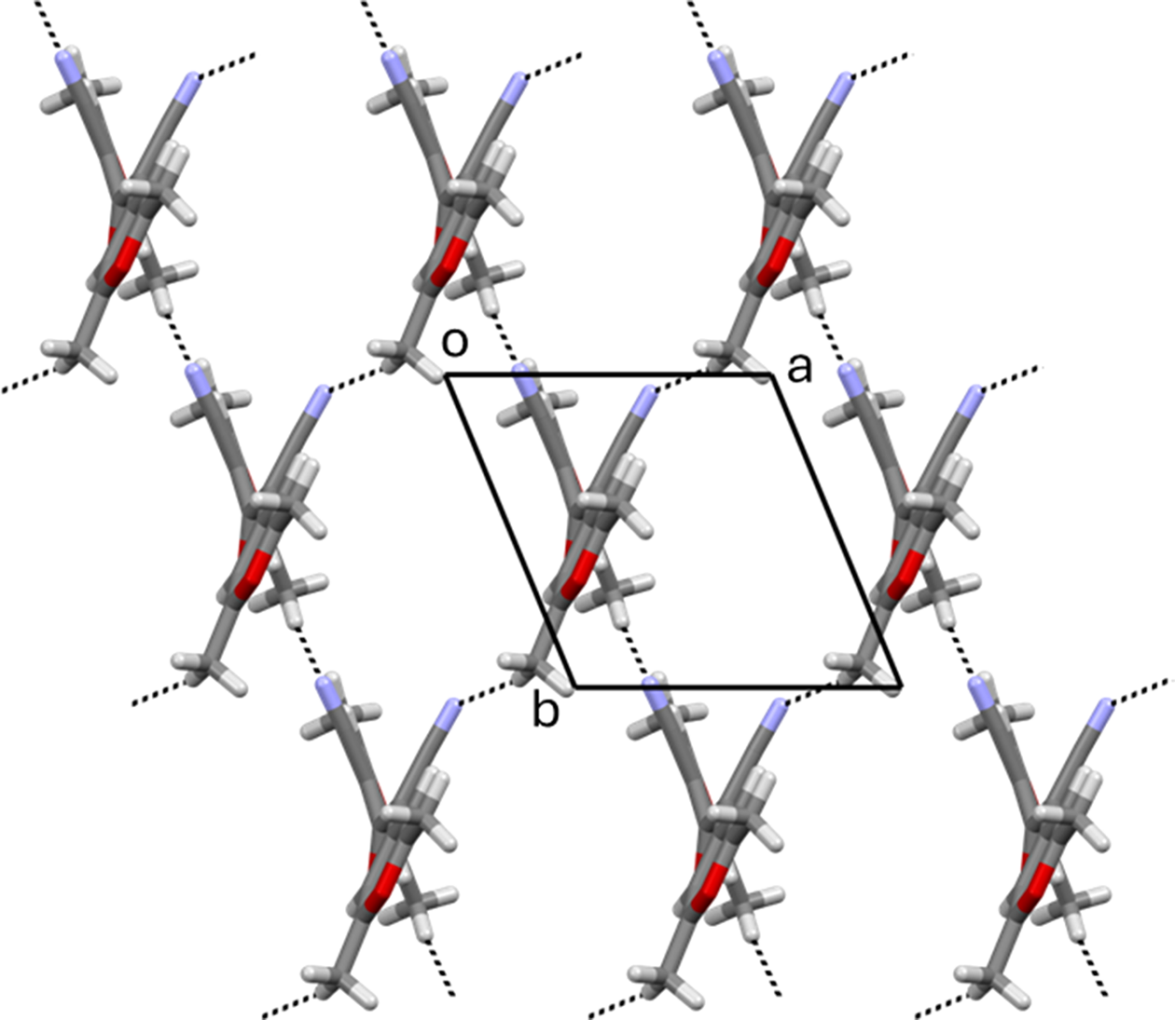
The crystal packing of **CC**, viewed down *c*.

Each mol­ecule of **CC** is involved in the formation of two chains, through weak C—H⋯N hy­dro­gen bonds (Table 1 ▸ and Fig. 2 ▸). One chain is parallel to the [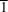
10] direction (C6—H6*A*⋯N1) and the other is parallel to [010] (C18—H18*A*⋯N2). In this way, an (001) bidimensional network of weakly hy­dro­gen-bonded mol­ecules is formed. The crystal packing is further consolidated by a weak C—H⋯O hy­dro­gen bond involving the carbonyl O atom as the acceptor (C19—H19*A*⋯O2).

## 4. Hirshfeld surface analysis

**Figure 3:**
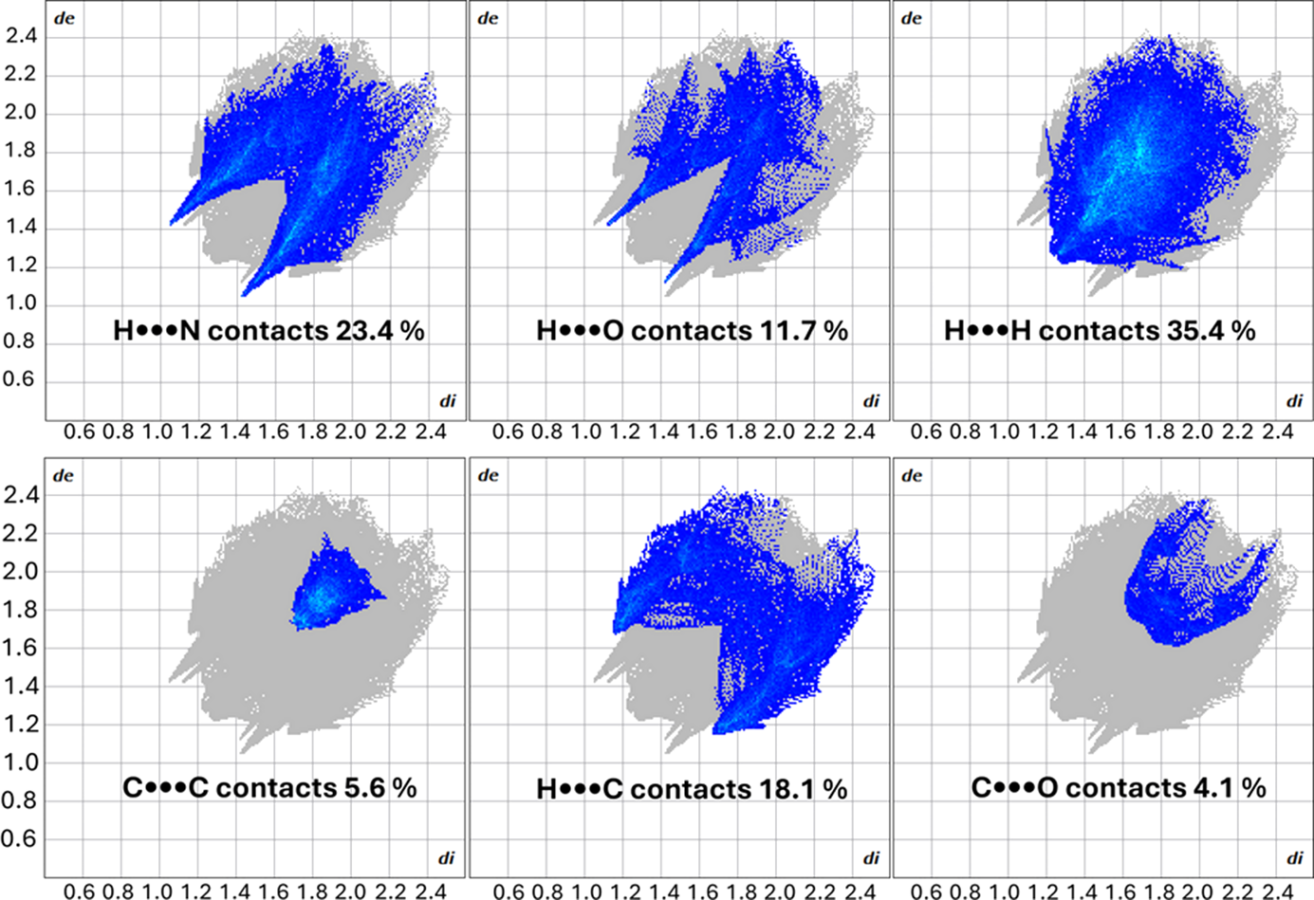
Fingerprint plots for the inter­molecular H⋯N, H⋯O, H⋯H, C⋯C, H⋯C and C⋯O contacts in the crystal structure of **CC**. All other inter­actions contribute less than 1%.

Hirshfeld surfaces were generated using the standard pro­molecule electron density based on spherical atomic electron densities, following the original definition of Hirshfeld partitioning of the crystal electron density (Spackman *et al.*, 2021 ▸). The *d*_norm_ surfaces were mapped over the range typically used for organic mol­ecular crystals (−0.5 to +1.5 Å). Distances *d*_i_ (inter­nal, from the surface to the nearest atom inside the surface) and *d*_e_ (external, to the nearest atom outside the surface) were com­puted for each surface point, and used to generate the corresponding two-dimensional fingerprint plots (*d*_e_*versus d*_i_), shown in Fig. 3 ▸, that summarize all inter­molecular contacts around the reference mol­ecule.

A distinctive feature is represented by the two spikes at *d*_i_ + *d*_e_ = 2.5 Å, pointing to the lower left of the plots and sym­metrically disposed with respect to the diagonal. They correspond to the C—H⋯N≡C weak hy­dro­gen-bonding inter­­actions. The two more inter­nal symmetrical spikes at *d*_i_ + *d*_e_ = 2.54 Å correspond instead to weak C—H⋯O=C hy­dro­gen bonding. The most abundant contacts are H⋯H, as expected. The green area centred at about (*d*_i_, *d*_e_) = (1.8, 1.8), corresponds to π–π stacking contacts.

## 5. Database survey

We searched the Cambridge Structural Database (CSD, Version 2025.6.0; Groom *et al.*, 2016 ▸) for structures similar to the title com­pound. In particular, we searched for structures containing the fragment com­posed of a central carbonyl group bonded to two C—CN groups. In the search, we applied the filters ‘no ions’ and ‘only organics’. Three hits were found [CSD refcodes AXOQOU and AXOQUA (Shiraki *et al.*, 2011 ▸), and CIFFAY (Medici *et al.*, 1984 ▸)], but none is really analogous to the title com­pound. In fact, in the three hits, the C atoms adjacent to the carbonyl group and bearing the cyano groups are *sp*^3^-hybridized. By releasing the filters, three additional hits were found [CSD refcodes PTCYPO (Klewe, 1971 ▸), VELPAE (de Oliveira *et al.*, 2006 ▸) and VODLIK (Atmani *et al.*, 2008 ▸)]. In PTCYPO and VELPAE, which are ionic, one C atom adjacent to the carbonyl group is *sp*^3^-hybridized and bears one CN group, while the other bears two CN groups and is anionic. In VODLIK, each C atom adjacent to the carbonyl bears two cyano groups and is anionic, with the CN groups coordinated to Cu^II^ ions in a coordination polymer.

## 6. Synthesis and crystallization

### 6.1. Synthesis

2,6-Dimethyl-γ-pyrone (11.0 g, 88.6 mmol) and cyano­acetic acid (9.0 g, 106 mmol) were dissolved in 30 ml of acetic anhydride, and a few drops of piperidine were added. The solution was refluxed, under nitro­gen flux, overnight. A brown solid formed during the reaction that, after the system was cooled to room tem­per­a­ture, was recovered by filtration. The recovered solid was washed in 100 ml of methanol and filtered again. Pure com­pound **CC** was obtained (yield 35%). Re­crystallization from hot di­methyl­formamide (DMF) solution afforded orange plates suitable for single-crystal X-ray analysis. ^1^H NMR (CDCl_3_, 400 MHz): δ (ppm) 7.63 (*s*, 1H), 6.67 (*s*, 1H), 2.32 (*s*, 3H), 2.29 (*s*, 3H). ^13^C NMR (CDCl_3_, 100 MHz): δ (ppm) 184.0, 162.8, 162.1, 153.8, 119.0, 90.1, 19.9.

### 6.2. Mechanism of reaction

The central bis-cyano­acetone portion of **CC** can be traced back to two cyano­acetic acid mol­ecules with the –COOH group of one cyano­acetic acid becoming the carbonyl group of **CC**, and the other one is lost, possibly as CO_2_, during the process.

**Figure 4:**
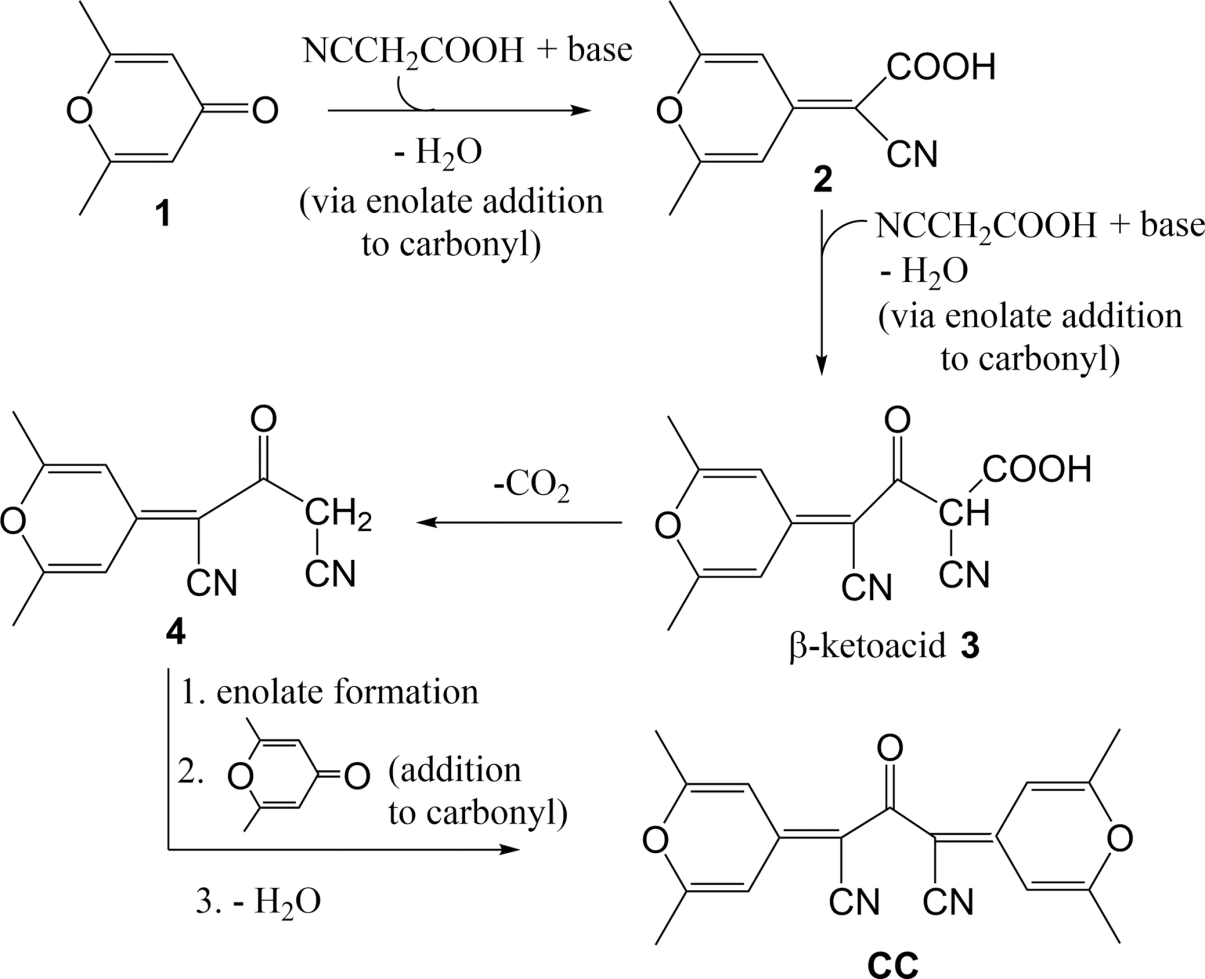
Possible reaction mechanism leading to **CC**.

Based on this, a plausible mechanistic hypothesis is shown in Fig. 4 ▸. In particular, attack of the enolate derived from cy­ano­acetic acid on the carbonyl group of 2,6-dimethyl-γ-py­rone (**1**), followed by loss of water gives cyano­acid **2**, which was the expected product. Attack of the enolate formed from a second cyano­acetic acid on the carbonyl group of the carb­oxy­lic acid function of **2**, and water loss, then follows giving β-ketoacid **3**. It is well known that β-ketoacids are prone to deca­rboxylation. Thus, deca­rboxylation of **3** gives β-keto­nitrile **4**, the enolate form of which attacks the carbonyl group of a second mol­ecule of **1**, eventually giving the final product **CC**, after water elimination. It is likely that the process is driven to com­pletion by the extended conjugation of the final product **CC** and by its precipitation from the reaction medium.

## 7. Refinement

Crystal data, data collection and structure refinement details are summarized in Table 2 ▸. H atoms were placed in calculated positions and refined using the riding model, with C—H distances of 0.93 Å for C*sp*^2^ and 0.96 Å for C*sp*^3^ atoms. The constraint *U*_iso_(H) = 1.2*U*_eq_(C) or 1.5*U*_eq_(methyl C) was applied in all cases.

## Supplementary Material

Crystal structure: contains datablock(s) I, global. DOI: 10.1107/S2056989026000587/hb8188sup1.cif

Structure factors: contains datablock(s) I. DOI: 10.1107/S2056989026000587/hb8188Isup2.hkl

Supporting information file. DOI: 10.1107/S2056989026000587/hb8188Isup3.cml

CCDC reference: 2524654

Additional supporting information:  crystallographic information; 3D view; checkCIF report

## Figures and Tables

**Table 1:** Hydrogen-bond geometry (Å, °)

*D*—H⋯*A*	*D*—H	H⋯*A*	*D*⋯*A*	*D*—H⋯*A*
C4—H4⋯O2	0.93	2.26	2.876 (2)	123
C17—H17⋯O2	0.93	2.33	2.908 (2)	120
C6—H6*A*⋯N1^i^	0.96	2.62	3.557 (3)	166
C18—H18*A*⋯N2^ii^	0.96	2.48	3.425 (3)	169
C19—H19*A*⋯O2^iii^	0.96	2.55	3.446 (3)	155

Symmetry codes: (i) 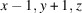; (ii) 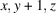; (iii) 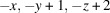.

**Table 2:** Experimental details

Crystal data	
Chemical formula	C_19_H_16_N_2_O_3_
*M*_r_	320.34
Crystal system, space group	Triclinic, *P* 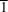
Temperature (K)	294
*a*, *b*, *c* (Å)	7.7530 (7), 8.0810 (14), 14.869 (2)
α, β, γ (°)	74.275 (13), 75.185 (12), 64.741 (13)
*V* (Å^3^)	800.4 (2)
*Z*	2
Radiation type	Mo *K*α
μ (mm^−1^)	0.09
Crystal size (mm)	0.40 × 0.40 × 0.10

Data collection	
Diffractometer	Bruker–Nonius KappaCCD
Absorption correction	Multi-scan (*SADABS*; Bruker, 2000 ▸)
*T*_min_, *T*_max_	0.950, 0.980
No. of measured, independent and observed [*I* > 2σ(*I*)] reflections	8620, 3584, 2683
*R*_int_	0.037
(sin θ/λ)_max_ (Å^−1^)	0.650

Refinement	
*R*[*F*^2^ > 2σ(*F*^2^)], *wR*(*F*^2^), *S*	0.051, 0.140, 1.04
No. of reflections	3584
No. of parameters	221
H-atom treatment	H-atom parameters constrained
Δρ_max_, Δρ_min_ (e Å^−3^)	0.21, −0.23

Computer programs: *COLLECT* (Nonius, 1999 ▸), *DIRAX*/*LSQ* (Duisenberg *et al.*, 2000 ▸), *EVALCCD* (Duisenberg *et al.*, 2003 ▸), *SIR97* (Altomare *et al.*, 1999 ▸), *SHELXL2019* (Sheldrick, 2015 ▸), *ORTEP-3 for Windows* (Farrugia, 2012 ▸) and *Mercury* (Macrae *et al.*, 2020 ▸).

## supplementary crystallographic information

### 2,4-Bis(2,6-dimethyl-4H-pyran-4-ylidene)-3-oxopentanedinitrile Crystal data

**Table:**

C_19_H_16_N_2_O_3_	*Z* = 2
*M**_r_* = 320.34	*F*(000) = 336
Triclinic, *P*1	*D*_x_ = 1.329 Mg m^−^^3^
*a* = 7.7530 (7) Å	Mo *K*α radiation, λ = 0.71073 Å
*b* = 8.0810 (14) Å	Cell parameters from 140 reflections
*c* = 14.869 (2) Å	θ = 3.1–23.8°
α = 74.275 (13)°	µ = 0.09 mm^−^^1^
β = 75.185 (12)°	*T* = 294 K
γ = 64.741 (13)°	Plate, orange
*V* = 800.4 (2) Å^3^	0.40 × 0.40 × 0.10 mm

### 2,4-Bis(2,6-dimethyl-4H-pyran-4-ylidene)-3-oxopentanedinitrile Data collection

**Table:**

Bruker–Nonius KappaCCD diffractometer	3584 independent reflections
Radiation source: normal-focus sealed tube	2683 reflections with *I* > 2σ(*I*)
Graphite monochromator	*R*_int_ = 0.037
Detector resolution: 9 pixels mm^-1^	θ_max_ = 27.5°, θ_min_ = 3.1°
CCD rotation images, thick slices scans	*h* = −10→9
Absorption correction: multi-scan (*SADABS*; Bruker, 2000)	*k* = −10→10
*T*_min_ = 0.950, *T*_max_ = 0.980	*l* = −19→19
8620 measured reflections	

### 2,4-Bis(2,6-dimethyl-4H-pyran-4-ylidene)-3-oxopentanedinitrile Refinement

**Table:**

Refinement on *F*^2^	Primary atom site location: structure-invariant direct methods
Least-squares matrix: full	Secondary atom site location: difference Fourier map
*R*[*F*^2^ > 2σ(*F*^2^)] = 0.051	Hydrogen site location: inferred from neighbouring sites
*wR*(*F*^2^) = 0.140	H-atom parameters constrained
*S* = 1.04	*w* = 1/[σ^2^(*F*_o_^2^) + (0.0634*P*)^2^ + 0.2356*P*] where *P* = (*F*_o_^2^ + 2*F*_c_^2^)/3
3584 reflections	(Δ/σ)_max_ < 0.001
221 parameters	Δρ_max_ = 0.21 e Å^−^^3^
0 restraints	Δρ_min_ = −0.23 e Å^−^^3^

### 2,4-Bis(2,6-dimethyl-4H-pyran-4-ylidene)-3-oxopentanedinitrile Special details

**Table:**

Geometry. All esds (except the esd in the dihedral angle between two l.s. planes) are estimated using the full covariance matrix. The cell esds are taken into account individually in the estimation of esds in distances, angles and torsion angles; correlations between esds in cell parameters are only used when they are defined by crystal symmetry. An approximate (isotropic) treatment of cell esds is used for estimating esds involving l.s. planes.

### 2,4-Bis(2,6-dimethyl-4H-pyran-4-ylidene)-3-oxopentanedinitrile Fractional atomic coordinates and isotropic or equivalent isotropic displacement parameters (Å^2^)

**Table:**

	*x*	*y*	*z*	*U*_iso_*/*U*_eq_	
C1	0.2922 (3)	0.4830 (3)	0.37307 (11)	0.0388 (4)	
C2	0.3497 (2)	0.3930 (2)	0.45670 (11)	0.0367 (4)	
H2	0.450561	0.276722	0.460459	0.044*	
C3	0.2594 (2)	0.4718 (2)	0.54069 (10)	0.0318 (4)	
C4	0.1080 (2)	0.6506 (2)	0.52650 (11)	0.0344 (4)	
H4	0.041867	0.709707	0.577950	0.041*	
C5	0.0581 (2)	0.7362 (2)	0.44048 (11)	0.0352 (4)	
C6	−0.0906 (3)	0.9234 (3)	0.41601 (14)	0.0490 (5)	
H6A	−0.159310	0.971978	0.472773	0.073*	
H6B	−0.179447	0.915454	0.384245	0.073*	
H6C	−0.029885	1.004411	0.375101	0.073*	
C7	0.3683 (3)	0.4138 (3)	0.28208 (13)	0.0591 (6)	
H7A	0.472739	0.294551	0.290938	0.089*	
H7B	0.414138	0.500210	0.235151	0.089*	
H7C	0.267133	0.402004	0.261421	0.089*	
C8	0.3153 (2)	0.3736 (2)	0.62836 (11)	0.0336 (4)	
C9	0.4731 (3)	0.1983 (3)	0.63024 (11)	0.0400 (4)	
C10	0.2283 (3)	0.4439 (2)	0.71759 (11)	0.0368 (4)	
C11	0.2435 (2)	0.3048 (2)	0.80721 (11)	0.0337 (4)	
C12	0.2411 (3)	0.1301 (3)	0.80563 (11)	0.0394 (4)	
C13	0.2435 (2)	0.3373 (2)	0.89399 (11)	0.0311 (3)	
C14	0.2535 (2)	0.1961 (2)	0.97809 (11)	0.0350 (4)	
H14	0.262779	0.080200	0.973095	0.042*	
C15	0.2500 (2)	0.2248 (2)	1.06311 (11)	0.0346 (4)	
C16	0.2392 (2)	0.5267 (2)	0.99654 (11)	0.0336 (4)	
C17	0.2370 (2)	0.5076 (2)	0.90996 (11)	0.0330 (4)	
H17	0.231191	0.606318	0.859428	0.040*	
C18	0.2362 (3)	0.6932 (3)	1.02192 (13)	0.0455 (4)	
H18A	0.224286	0.790773	0.967157	0.068*	
H18B	0.354003	0.662126	1.044252	0.068*	
H18C	0.128336	0.734558	1.070808	0.068*	
C19	0.2482 (3)	0.0926 (3)	1.15466 (12)	0.0511 (5)	
H19A	0.127048	0.141621	1.194979	0.077*	
H19B	0.351677	0.076023	1.184774	0.077*	
H19C	0.264671	−0.025091	1.143354	0.077*	
N1	0.6038 (3)	0.0598 (3)	0.62864 (12)	0.0595 (5)	
N2	0.2324 (3)	−0.0090 (2)	0.80821 (11)	0.0579 (5)	
O1	0.14832 (18)	0.65436 (17)	0.36313 (8)	0.0407 (3)	
O2	0.1467 (2)	0.61046 (18)	0.71935 (9)	0.0600 (4)	
O3	0.24437 (17)	0.38884 (16)	1.07394 (8)	0.0379 (3)	

### 2,4-Bis(2,6-dimethyl-4H-pyran-4-ylidene)-3-oxopentanedinitrile Atomic displacement parameters (Å^2^)

**Table:**

	*U*^11^	*U*^22^	*U*^33^	*U*^12^	*U*^13^	*U*^23^
C1	0.0468 (10)	0.0461 (10)	0.0275 (8)	−0.0229 (8)	−0.0015 (7)	−0.0089 (7)
C2	0.0410 (9)	0.0405 (9)	0.0280 (8)	−0.0168 (7)	−0.0013 (7)	−0.0076 (7)
C3	0.0400 (9)	0.0373 (9)	0.0255 (8)	−0.0233 (7)	−0.0028 (6)	−0.0056 (6)
C4	0.0483 (9)	0.0356 (9)	0.0256 (8)	−0.0218 (8)	−0.0038 (6)	−0.0078 (6)
C5	0.0477 (9)	0.0360 (9)	0.0289 (8)	−0.0218 (7)	−0.0062 (7)	−0.0070 (7)
C6	0.0687 (13)	0.0397 (11)	0.0392 (10)	−0.0174 (9)	−0.0189 (9)	−0.0045 (8)
C7	0.0714 (14)	0.0700 (14)	0.0280 (9)	−0.0185 (11)	−0.0017 (9)	−0.0171 (9)
C8	0.0413 (9)	0.0355 (9)	0.0262 (8)	−0.0174 (7)	−0.0048 (6)	−0.0052 (6)
C9	0.0452 (10)	0.0479 (11)	0.0250 (8)	−0.0186 (9)	−0.0028 (7)	−0.0050 (7)
C10	0.0489 (10)	0.0365 (10)	0.0268 (8)	−0.0158 (8)	−0.0094 (7)	−0.0066 (7)
C11	0.0411 (9)	0.0338 (9)	0.0250 (8)	−0.0124 (7)	−0.0047 (6)	−0.0073 (6)
C12	0.0548 (10)	0.0419 (10)	0.0214 (8)	−0.0196 (8)	−0.0036 (7)	−0.0063 (7)
C13	0.0303 (8)	0.0332 (8)	0.0253 (7)	−0.0077 (6)	−0.0036 (6)	−0.0065 (6)
C14	0.0417 (9)	0.0319 (9)	0.0281 (8)	−0.0100 (7)	−0.0053 (6)	−0.0077 (7)
C15	0.0362 (8)	0.0335 (9)	0.0294 (8)	−0.0081 (7)	−0.0066 (6)	−0.0059 (7)
C16	0.0334 (8)	0.0368 (9)	0.0295 (8)	−0.0117 (7)	−0.0045 (6)	−0.0079 (7)
C17	0.0357 (8)	0.0347 (9)	0.0258 (8)	−0.0118 (7)	−0.0039 (6)	−0.0051 (6)
C18	0.0573 (11)	0.0469 (11)	0.0400 (10)	−0.0242 (9)	−0.0079 (8)	−0.0128 (8)
C19	0.0711 (13)	0.0456 (11)	0.0282 (9)	−0.0160 (10)	−0.0118 (8)	−0.0007 (8)
N1	0.0545 (10)	0.0586 (11)	0.0410 (9)	−0.0029 (9)	−0.0031 (7)	−0.0072 (8)
N2	0.0952 (14)	0.0525 (11)	0.0367 (9)	−0.0405 (10)	−0.0071 (8)	−0.0083 (7)
O1	0.0550 (7)	0.0432 (7)	0.0253 (6)	−0.0199 (6)	−0.0073 (5)	−0.0063 (5)
O2	0.1017 (12)	0.0355 (8)	0.0332 (7)	−0.0097 (7)	−0.0227 (7)	−0.0082 (5)
O3	0.0483 (7)	0.0410 (7)	0.0248 (6)	−0.0157 (5)	−0.0086 (5)	−0.0068 (5)

### 2,4-Bis(2,6-dimethyl-4H-pyran-4-ylidene)-3-oxopentanedinitrile Geometric parameters (Å, º)

**Table:**

C1—C2	1.338 (2)	C10—C11	1.486 (2)
C1—O1	1.356 (2)	C11—C13	1.385 (2)
C1—C7	1.487 (2)	C11—C12	1.426 (2)
C2—C3	1.435 (2)	C12—N2	1.144 (2)
C2—H2	0.9300	C13—C17	1.437 (2)
C3—C8	1.397 (2)	C13—C14	1.440 (2)
C3—C4	1.423 (2)	C14—C15	1.337 (2)
C4—C5	1.345 (2)	C14—H14	0.9300
C4—H4	0.9300	C15—O3	1.360 (2)
C5—O1	1.3620 (19)	C15—C19	1.486 (2)
C5—C6	1.476 (3)	C16—C17	1.343 (2)
C6—H6A	0.9600	C16—O3	1.363 (2)
C6—H6B	0.9600	C16—C18	1.482 (2)
C6—H6C	0.9600	C17—H17	0.9300
C7—H7A	0.9600	C18—H18A	0.9600
C7—H7B	0.9600	C18—H18B	0.9600
C7—H7C	0.9600	C18—H18C	0.9600
C8—C9	1.421 (2)	C19—H19A	0.9600
C8—C10	1.470 (2)	C19—H19B	0.9600
C9—N1	1.147 (2)	C19—H19C	0.9600
C10—O2	1.223 (2)		
			
C2—C1—O1	121.73 (15)	C13—C11—C12	117.26 (14)
C2—C1—C7	126.75 (18)	C13—C11—C10	124.94 (15)
O1—C1—C7	111.51 (15)	C12—C11—C10	117.57 (14)
C1—C2—C3	121.56 (16)	N2—C12—C11	176.67 (19)
C1—C2—H2	119.2	C11—C13—C17	125.09 (14)
C3—C2—H2	119.2	C11—C13—C14	121.37 (15)
C8—C3—C4	124.46 (14)	C17—C13—C14	113.53 (14)
C8—C3—C2	121.12 (15)	C15—C14—C13	122.65 (15)
C4—C3—C2	114.39 (14)	C15—C14—H14	118.7
C5—C4—C3	121.60 (15)	C13—C14—H14	118.7
C5—C4—H4	119.2	C14—C15—O3	121.20 (14)
C3—C4—H4	119.2	C14—C15—C19	126.78 (16)
C4—C5—O1	121.51 (16)	O3—C15—C19	112.01 (14)
C4—C5—C6	126.76 (16)	C17—C16—O3	122.46 (15)
O1—C5—C6	111.71 (14)	C17—C16—C18	126.40 (16)
C5—C6—H6A	109.5	O3—C16—C18	111.14 (14)
C5—C6—H6B	109.5	C16—C17—C13	121.18 (15)
H6A—C6—H6B	109.5	C16—C17—H17	119.4
C5—C6—H6C	109.5	C13—C17—H17	119.4
H6A—C6—H6C	109.5	C16—C18—H18A	109.5
H6B—C6—H6C	109.5	C16—C18—H18B	109.5
C1—C7—H7A	109.5	H18A—C18—H18B	109.5
C1—C7—H7B	109.5	C16—C18—H18C	109.5
H7A—C7—H7B	109.5	H18A—C18—H18C	109.5
C1—C7—H7C	109.5	H18B—C18—H18C	109.5
H7A—C7—H7C	109.5	C15—C19—H19A	109.5
H7B—C7—H7C	109.5	C15—C19—H19B	109.5
C3—C8—C9	117.56 (14)	H19A—C19—H19B	109.5
C3—C8—C10	124.29 (15)	C15—C19—H19C	109.5
C9—C8—C10	118.07 (14)	H19A—C19—H19C	109.5
N1—C9—C8	177.40 (19)	H19B—C19—H19C	109.5
O2—C10—C8	122.05 (15)	C1—O1—C5	119.20 (13)
O2—C10—C11	120.33 (15)	C15—O3—C16	118.93 (12)
C8—C10—C11	117.62 (14)		
			
O1—C1—C2—C3	1.2 (3)	C12—C11—C13—C17	−177.06 (15)
C7—C1—C2—C3	−177.46 (17)	C10—C11—C13—C17	−2.8 (3)
C1—C2—C3—C8	177.42 (15)	C12—C11—C13—C14	4.0 (2)
C1—C2—C3—C4	−0.5 (2)	C10—C11—C13—C14	178.25 (15)
C8—C3—C4—C5	−178.45 (15)	C11—C13—C14—C15	−178.69 (15)
C2—C3—C4—C5	−0.7 (2)	C17—C13—C14—C15	2.3 (2)
C3—C4—C5—O1	1.0 (2)	C13—C14—C15—O3	−2.7 (2)
C3—C4—C5—C6	−177.63 (16)	C13—C14—C15—C19	176.24 (16)
C4—C3—C8—C9	−178.33 (15)	O3—C16—C17—C13	−1.1 (2)
C2—C3—C8—C9	4.0 (2)	C18—C16—C17—C13	179.01 (15)
C4—C3—C8—C10	−1.6 (2)	C11—C13—C17—C16	−179.40 (15)
C2—C3—C8—C10	−179.27 (15)	C14—C13—C17—C16	−0.4 (2)
C3—C8—C10—O2	−23.9 (3)	C2—C1—O1—C5	−0.9 (2)
C9—C8—C10—O2	152.77 (18)	C7—C1—O1—C5	178.00 (15)
C3—C8—C10—C11	156.50 (15)	C4—C5—O1—C1	−0.3 (2)
C9—C8—C10—C11	−26.8 (2)	C6—C5—O1—C1	178.58 (15)
O2—C10—C11—C13	−26.7 (3)	C14—C15—O3—C16	1.0 (2)
C8—C10—C11—C13	152.82 (16)	C19—C15—O3—C16	−178.02 (14)
O2—C10—C11—C12	147.49 (18)	C17—C16—O3—C15	0.9 (2)
C8—C10—C11—C12	−33.0 (2)	C18—C16—O3—C15	−179.25 (14)

### 2,4-Bis(2,6-dimethyl-4H-pyran-4-ylidene)-3-oxopentanedinitrile Hydrogen-bond geometry (Å, º)

**Table:**

*D*—H···*A*	*D*—H	H···*A*	*D*···*A*	*D*—H···*A*
C4—H4···O2	0.93	2.26	2.876 (2)	123
C17—H17···O2	0.93	2.33	2.908 (2)	120
C6—H6*A*···N1^i^	0.96	2.62	3.557 (3)	166
C18—H18*A*···N2^ii^	0.96	2.48	3.425 (3)	169
C19—H19*A*···O2^iii^	0.96	2.55	3.446 (3)	155

Symmetry codes: (i) *x*−1, *y*+1, *z*; (ii) *x*, *y*+1, *z*; (iii) −*x*, −*y*+1, −*z*+2.
